# Signalling dynamics, cell decisions, and homeostatic control in health and disease

**DOI:** 10.1016/j.ceb.2022.01.011

**Published:** 2022-04

**Authors:** Pablo Oriol Valls, Alessandro Esposito

**Affiliations:** 1 MRC Cancer Unit, University of Cambridge, Cambridge, CB2 0XZ, United Kingdom; 2 Centre for Genome Engineering and Maintenance, College of Health, Medicine and Life Sciences, Brunel University London, Uxbridge, UB8 3PH, United Kingdom

## Abstract

Cell signalling engenders cells with the capability to receive and process information from the intracellular and extracellular environments, trigger and execute biological responses, and communicate with each other. Ultimately, cell signalling is responsible for maintaining homeostasis at the cellular, tissue and systemic level. For this reason, cell signalling is a topic of intense research efforts aimed to elucidate how cells coordinate transitions between states in developing and adult organisms in physiological and pathological conditions. Here, we review current knowledge of how cell signalling operates at multiple spatial and temporal scales, focusing on how single-cell analytical techniques reveal mechanisms underpinning cell-to-cell variability, signalling plasticity, and collective cellular responses.

## Introduction

In 1854, physiologist Claud Bernard postulated that the self-regulation of their internal environment is a pre-condition for the existence of complex systems such as the human body. Sixty years later, Walter B. Cannon defines this fundamental feature of living organisms as *homeostasis* [1]. Despite its etymology (*i.e.*, keeping similar), homeostasis is a highly dynamic process during which only a few internal physicochemical properties are maintained within a narrow range, that is, they are homeostatically controlled. In complex organisms, homeostatic control is critical at the system, tissue, and cellular levels. For example, the brain, lungs, kidneys, and red blood cells contribute to pH regulation in plasma; adult stem cells regenerate tissues to maintain their integrity; cells maintain the basal concentrations of Ca^2+^, Na^+^, and K^+^, within narrow ranges.

**Figure 1:**
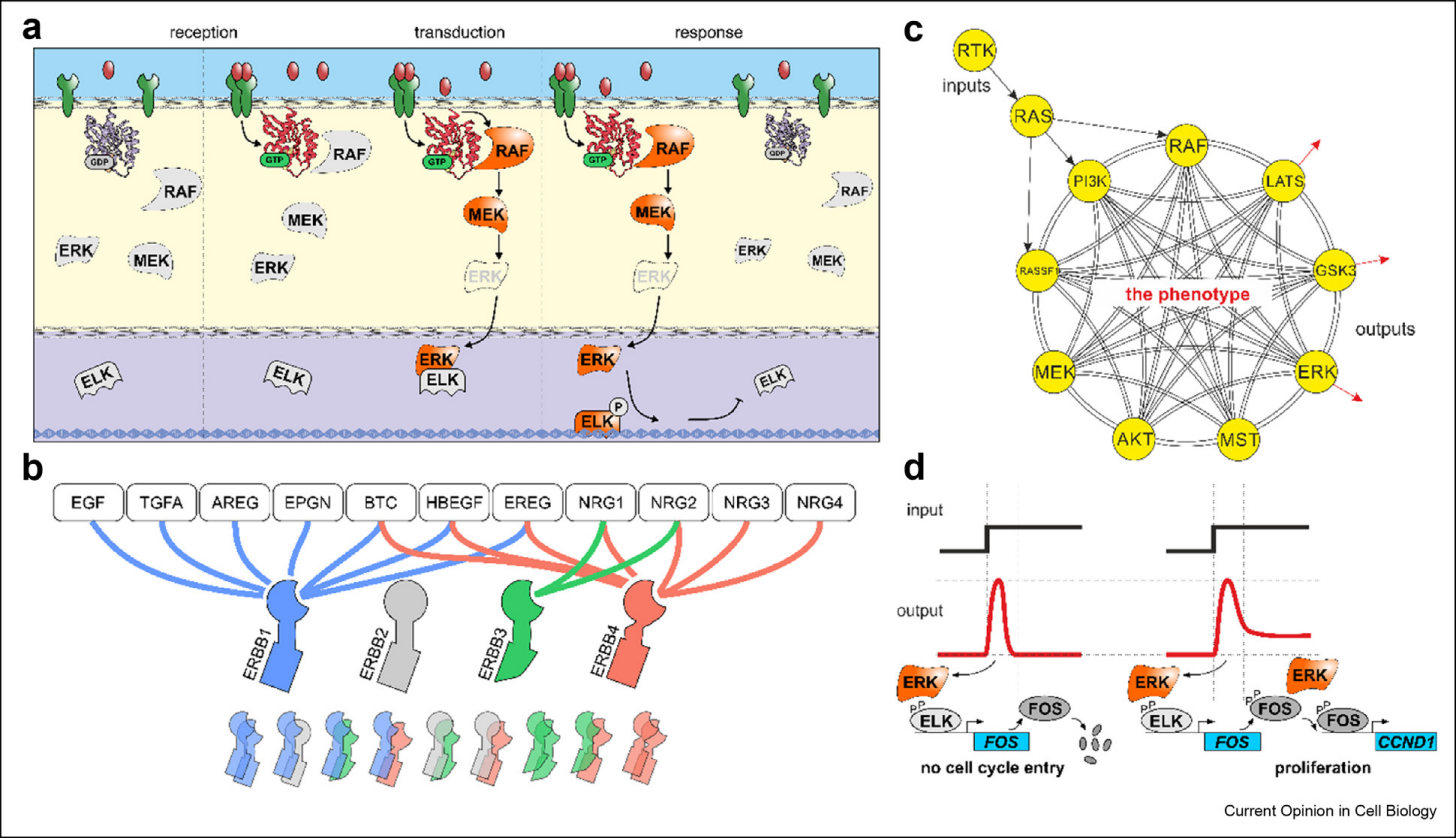
**Cell *signalling. a)****Diagrammatic representation of signal reception, transduction, and response, exemplified by ERBB receptor tyrosine kinases and MAPK signalling. Ligand binding triggers receptor dimerisation at the plasma membrane, leading to their cytoplasmic domain phosphorylation and the recruitment of signalling complexes (not shown). ERBB activation then facilitates the exchange of GDP for GTP in small GTPases (e.g. RAS proteins), inducing the recruitment of effector kinases. Here, we depict one of the several pathways that amplify and relay the initiating signal to the nucleus, the MAPK pathway (RAF→MEK→ERK). In the nucleus, ERK phosphorylates ELK leading to transcription of target genes, biological response, and termination of the originating signal.****b)****Diagrammatic representation of ERBB ligand reception. ERBB1 (also known as EGFR) binds the epidermal growth factor (EGF), transforming growth factor alpha (TGFA), amphiregulin (AREG), heparin-binding EGF-like growth factor (HBEGF), betacellulin (BTC), epigen (EPGN) and epiregulin (EREG). ERBB2 has no ligand but can heterodimerise with other ERBB receptors. ERBB3 has very low kinase activity on its own but forms active dimers upon binding to neuregulins (NRG1/2). ERBB4 binds to several ligands of ERBB1/3, and NRG3/4.****c)****Once a molecular event (ligand binding) is transduced to a biochemical signal (RTK phosphorylation), a network of networks (here we depict MAPK, PI3K, and hippo pathways) further process the biochemical signal in space and time to trigger the most appropriate biological response.****d)****Signalling dynamics favour the activation of specific transcriptional programs depending on the stability of target transcripts and proteins. For example, ELK induce the transcription of FOS that is rapidly degraded. However, if ERK activation is sustained, ERK phosphorylate also FOS, leading to proliferation through Cyclin D1 (CCDN1) expression* [14].

Homeostatic control relies on biochemical networks we refer to as *cell signalling,* which engender cells the capability to sense physicochemical cues, process information and execute the most appropriate biological responses (Figure 1a). Here, we highlight recent conceptual and methodological advancements that are helping us better understand molecular mechanisms underpinning homeostatic control. Moreover, we discuss how disease often stems from the corruption of cell signalling and the consequent deregulation of homeostasis.

## Signal reception

Cells sense many physicochemical cues with a diversified complement of signalling machinery. Figure 1a–b depicts ERBB receptor tyrosine kinases (RTK) as an instructive example for receptor-mediated signalling [2,3]. The ERBB receptor family comprises four transmembrane proteins ERBB1-4 that bind more than ten ligands with varying specificities (Figure 1b) [4]. Binding favours ligand-dependent homo- and hetero-dimerisation of the receptors, autophosphorylation of their cytoplasmic domains and engagement of effector proteins. Arguably, receptors do not just convert a molecular event (binding) to a biochemical signal that will be further relayed by signal transduction pathways. Different ERBB dimers exhibit both varying affinities for downstream effectors and different regulatory sites. Therefore, ERBB dimers establish distinct positive and negative feedbacks with signal transduction networks [5, 6, 7]. These interactions eventually result both in the spatial propagation of the signal [8,9] and into characteristic temporal dynamics that encode the identity, amplitude, and duration of the originating stimulus.

Cell signalling is also initiated intra-cellularly and without receptors, for example, in the DNA damage response [10]. There are several types of DNA lesions, each requiring specific repair mechanisms. For instance, double-strand breaks are detected by two protein complexes, namely, Ku (XRCC5 and XRCC6) and the MRN complex (MRE11, RAD50, and NBS1 also known as NBN); single-strand breaks are sensed by RPA proteins, TOPBP1 and ATRIP. Ku, MRN, and RPA/ATRIP recruit to the site of damage the three kinases DNA-PKcs (PRKDC), ATM and ATR, respectively. Active DNA-PKcs, ATM, and ATR then coordinate specific repair processes (*e.g.*, non-homologous end-joining, homologous recombination and Fanconi anaemia repair pathways, respectively). At the same time, DNA-PKcs, ATM, and ATR amplify the signal initiated by an individual DNA lesion both at the site of damage and towards other cell compartments leading to cell cycle arrest, or cell death in the presence of unresolved damage.

ERBB and DNA damage signalling are two very different examples of signal reception amongst a large variety of diverse signalling mechanisms. Both examples, however, illustrate how initiating events usually trigger the formation or activation of multi-molecular signalling platforms. These structures, nowadays more frequently but not universally referred to as signalosomes [11•, 12, 13], initiate signal transduction from sensors to downstream effector pathways, from sites of reception to different cellular locations, and integrate other modulatory inputs and feedbacks from downstream networks. Signal reception already embeds signal processing characteristics that are often poorly understood but critical in shaping downstream cellular responses.

## Signalling pathways and signalling dynamics

The realisation that spatiotemporal dynamics plays a fundamental role in cell signalling can be tracked back to the early discovery of the action potential in 1843 by Emil du Bois-Reymond [15]. Nerve conduction is indeed a specialised form of cell signalling. Neurons integrate numerous signals and encode information both in the amplitude of an action potential travelling along the axon and the frequency of a neuron's firing, that is*,* in the temporal dynamics. With the advent of the modern biochemistry of the 20th century, the importance of spatiotemporal dynamics in all tissues and at all scales became increasingly apparent, for example to explain morphogens [16] and cell cycle control [17]. ERBB signalling also exhibits distinctive spatiotemporal dynamics.

Differences in negative feedbacks in ERBB1–ERBB1 and ERBB3–ERBB4 heterodimers, for example, trigger transient or sustained activation of the mitogen-activated protein kinase (MAPK) pathway in response to EGF and NRG1, respectively (Figure 1a, c) [18]. Furthermore, cross-talk between signalling pathways permits cells to integrate different signals. For example, when stimulated with EGF, pheochromocytoma rat cells (PC-12) exhibit the characteristic transient response in extracellular regulated kinases (ERK1/2, also known as MAPK1/3) activity [19]. Instead, the nerve growth factor (NGF) binds the neurotrophic receptor tyrosine kinase 1 (NTRK1, also known as TrkA) and triggers sustained ERK activity. Differences in signalling dynamics also depend on the promiscuity of receptors with several signalling pathways and their cross-talk [20, 21, 22, 23, 24]. Santos et al. [21], for example, have reconstructed topological maps of MAPK signalling (see Figures 1c and 3a), demonstrating that either through direct or indirect interactions, different stimuli reshape network topologies encoding for distinct signalling dynamics, eventually resulting in different responses such as proliferation or differentiation of PC-12 cells in response to EGF and NGF, respectively.

**Figure 2:**
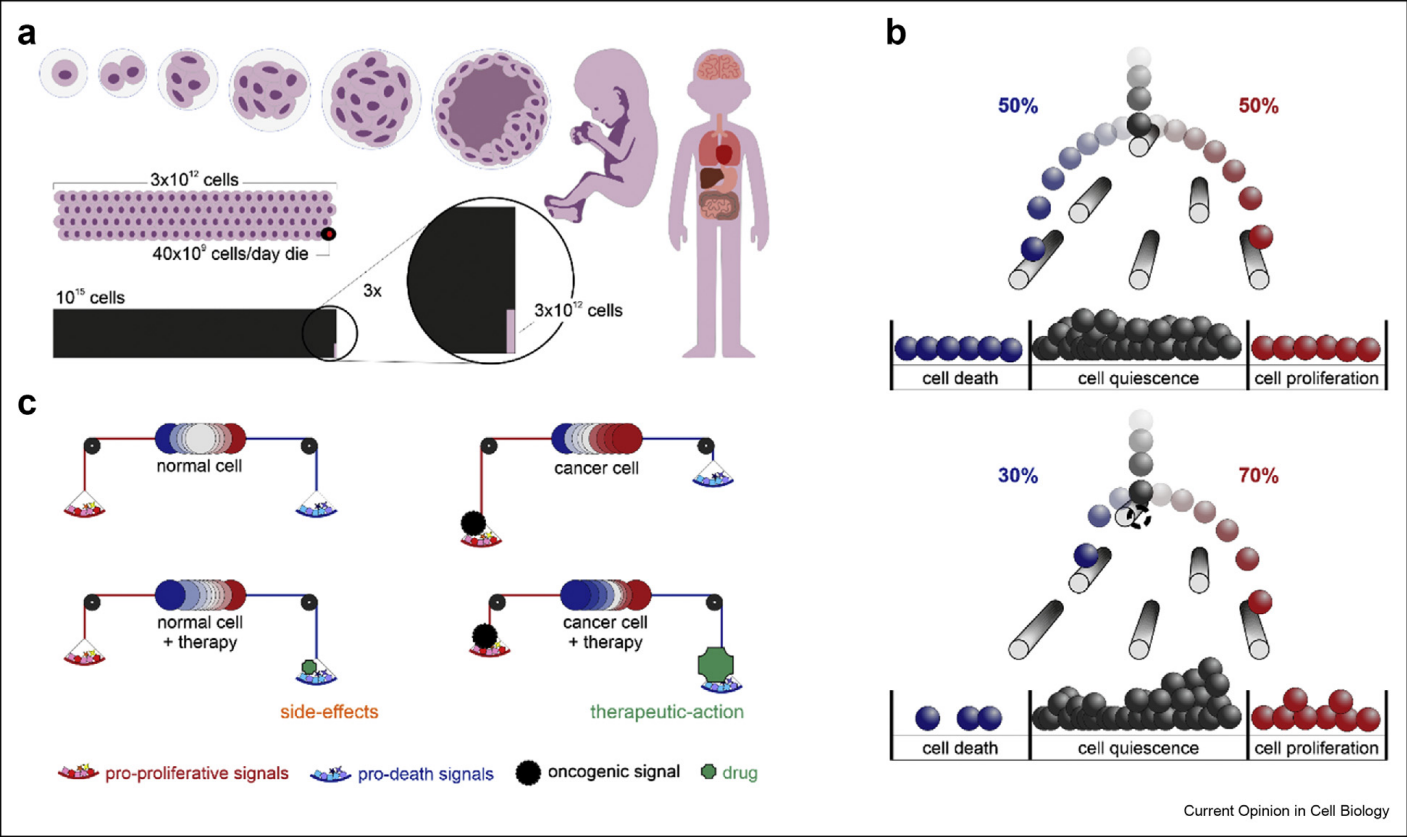
**Cell decisions and homeostasis. a)***Cellular decisions are essential during development and in adult tissues. Even excluding the hematopoietic system with its very high turnover, of the 3 trillion cells making up an adult human body, up to 40 billion die each day* [40]. *Turnover of cells varies significantly across tissues (being lowest in the brain and the highest in the gut and hematopoietic system); however, by the end of our lifespan, a human body might have replenished 1000 times more cells than the number of cells it has at any given time.****b)****Cell fates exhibit stochastic characteristics, here described by a Galton board. Cells are depicted by marbles falling onto pins (biochemical events). Each step is deterministic, but marbles eventually fall into a ‘cell fate’ box randomly following distributions determined by the geometrical configuration of the pins. Most cells are quiescent within a tissue, and an equal number of cells die and are born, on average, to maintain tissue homeostasis. Molecular cues favour one or the other cell fate, inducing tissue regeneration or leading to aberrant homeostasis.****c)****Genetically identical cells of the same type can thus exhibit vast cell-to-cell variability of non-genetic origin. Biochemical signals are then shifting the balance in the cellular population (in the analogy of the Galton board, the pins are moved), favouring homeostasis in healthy tissues or causing cell fate imbalances in disease (e.g. excess proliferation in cancer). Therapies aim to restore homeostasis in target tissue avoiding homeostatic disruption in others.*

**Figure 3:**
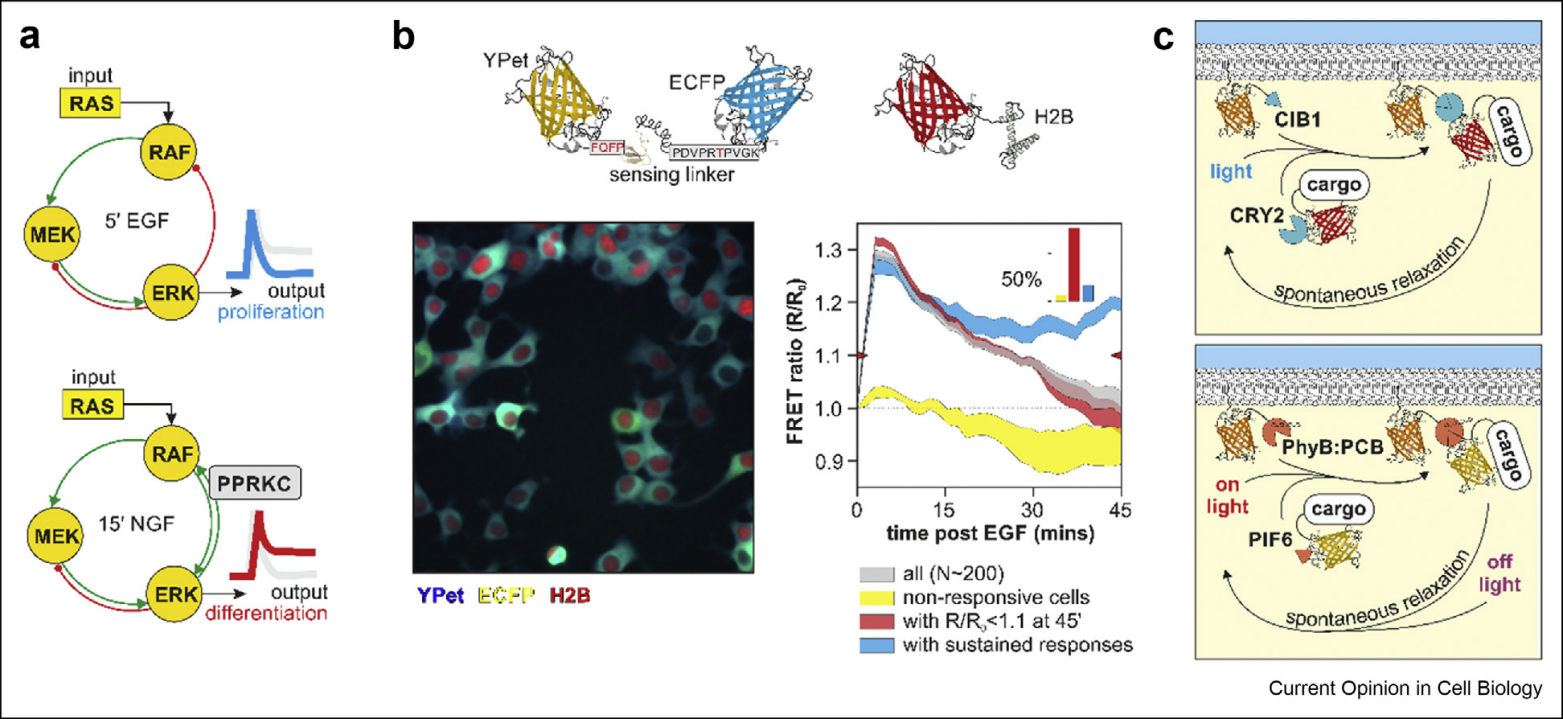
**Studying signalling dynamic*s in space and time. a)****Diagrammatic representation of MAPK network topology as determined by Santos et al.* [21]. *In response to EGF and NGF, ERK exhibit transient or sustained activation. Different dynamics are determined by distinct feedback mechanisms enacted by growth factor receptors, and result in different cell decisions (proliferation versus differentiation). However, each cell might respond with a different dynamic to the same stimulus. Panel****b)****shows MCF7 cells stably expressing the EKAREV ERK FRET-based sensor and a nuclear marker treated with* 100 ng/ml EGF*. The large majority of cells respond with the transient activation of ERK (red), similar to the average response (grey). However, ∼10% of cells either do not respond (yellow) or respond with sustained ERK signalling (blue). The non-responders and the sustained responders were classified as those cells that either do not cross the 1.*1 FRET *ratio threshold (red marks on the ordinate), or cells that raise above the marker initially but do not return below 1.1. Methods can be found in De* et al. [27] ***c)****Diagrammatic representation of optogenetic tools utilised to control cell signalling.*

The temporal evolution of a biochemical signal can take many different shapes, including periodic oscillations. For example, upon induction of double-strand breaks by γ-irradiation, several cell types exhibit periodic pulses of TP53 and DNA damage checkpoint kinase activities (*e.g.*, ATM and CHK1/2); however, single-strand breaks resulting from the repair of damage caused by UV radiation trigger a sustained response [10,25,26]. Pulsatile or oscillatory dynamics are triggered by the opposing effects of positive and negative feedbacks. For instance, the E3 ubiquitin ligase MDM2 is a transcriptional target of TP53 that induces its rapid degradation through the ubiquitin–proteasome system. Upon DNA damage triggering recruitment and activation of ATM, ATM phosphorylates and activates CHK kinases. ATM and CHK kinases then phosphorylate and stabilise TP53, which induces its negative regulator MDM2. Depending on the balance between stimuli and negative feedback, TP53 exhibits oscillatory dynamics that are backpropagated to ATM via the activity of WIP1 phosphatase. We have recently shown that these oscillations can also propagate to the MAPK pathway; additionally, MAPK signalling attenuates TP53 pulses when stimulated by NRG1 but not EGF because of the different ERK dynamics they trigger [27].

Therefore, a model of cell signalling where individual pathways are better understood as constituent parts of a ‘network of networks’ that process information and embed in their spatiotemporal dynamics the ‘code’ that regulates the transition between cellular states – or phenotypes (Figure 1c) is now emerging [28, 29, 30].

## Cellular response

Intense research efforts spanning cell biochemistry, computational biology and biophotonics are revealing how signalling dynamics regulates transitions between cellular states. The different stability of gene transcripts and proteins is a key molecular aspect for interpreting cell signalling. For example, the mRNA of immediate early genes (IEGs), *i.e.*, genes that do not require the *de novo* expression of accessory proteins for their transcription, can accumulate seconds or minutes after a triggering signal. Stable IEG transcripts accumulate over time and can induce expression of proteins even after a transient or pulsatile signal; instead, IEGs that are transcribed in mRNAs of short half-life require a sustained transcriptional activation to induce the expression of target proteins at biologically significant concentrations [31, 32, 33, 34].

Interestingly, under the effects of a prolonged stimulus, both short- and long-lived IEG transcripts are expressed. However, while the less stable mRNAs rapidly reach a steady-state concentration, long-lived IEGs can accumulate over time, thus encoding the duration of the originating stimulus in the amplitude of the mRNA response, [31]. The interplay between temporal patterns of signalling molecules and transcriptional machinery provides a robust and specific mechanism to induce different cellular responses (*e.g.*, survival, arrest, cell death, and differentiation).

Protein stabilities also play a fundamental role in decoding signalling dynamics into biological responses. For example (Figure 1d), ERK can induce the expression of the immediate early gene *FOS* by phosphorylating the transcriptional activator ELK [14]. FOS is a transcription factor that is rapidly degraded once expressed. However, when phosphorylated by ERK, FOS is stabilised and initiates the transcription of target genes such as cyclin D1 (*CCND1*). This and similar mechanisms permit cells to engage in the cell cycle only in the presence of non-spurious mitogenic cues, such as constant or properly-timed pulses of growth factors. Moreover, protein stabilisation can endow cells with the memory of past events also across generations, such as the occurrence of strongly mitogenic environments [14,35,36].

These mechanisms are not restricted to ERBB and mitogenic signalling. For example, Lahav et al.[25] have shown that TP53 oscillations maintain cells in a reversible cell cycle arrest conducive to DNA damage repair, while prolonged TP53 expression results in cell death. Both the dynamics of TP53 expression and that of target genes depends on a delicate balance of mRNA and protein stabilities. For example, while TP53 is a memoryless oscillator that promptly responds to DNA damage kinases such as ATM, its transcriptional target CDKN1A integrates TP53 dynamics over longer periods, maintaining a cell cycle arrest or eventually resulting in cell death [37].

Similar mechanisms are reported for several signalling pathways and transcriptional programs [26,38,39], suggesting that time-encoding of signals and transcriptional programs is a fundamental principle underpinning biological responses.

## Homeostasis, heterogeneity, and disease

In response to a stimulus, cell signalling thus coordinates molecular machinery (*e.g.*, ERBB-dependent cytoskeletal rearrangements, or DNA damage repair) with the transition between cellular states to maintain cell homeostasis and function. Similarly, by integrating cell-autonomous and non-cell-autonomous mechanisms, signalling determines cellular decisions that regulate the development of multi-cellular organisms and the homeostasis of adult tissues (Figure 2a). We can describe each step of these mechanisms deterministically; however, it is increasingly evident that the behaviour of cells within a population can be better described with stochastic models [41]. For example, the homeostasis of skin and the oesophageal epithelium is maintained by stem cells that, when proliferating, exhibit a given probability to either differentiate or self-renew. This probability is fine-tuned to ensure tissue renewal [42, 43, 44]. However, cell signalling corruption through cell-autonomous mechanisms or cell-to-cell communication can shift this balance, leading to diseases such as cancer (Figure 2b,c).

Therefore, a better understanding of these processes is critical to elucidate pathogenicity mechanisms and improve disease management. A notable example is provided by the difficulty in explaining the distribution of mutations that often occurs within signalling pathways that drive carcinogenesis in different tissues [45, 46, 47]. Even a given oncogenic driver (*e.g.,* KRAS) can exhibit distinct mutational patterns or gene amplifications that remain difficult to explain by tissue-dependent mutagenesis [48,49]. We can hypothesise that mutations result both in quantitative and qualitative alterations of cell signalling. Gene truncations, deletions, and inactivating mutations can remove essential nodes of biochemical networks; gene amplifications and activating mutations can drive excessive signalling. However, we now appreciate how these alterations might also exert more subtle effects on cell signalling, still corrupting signalling dynamics and drastically disrupting homeostatic control [23,30]. For example, similar mutations (*e.g.*, non-synonymous KRAS G12 mutations) result in similar yet quantitatively different signals [50,51]. Specific alterations might bring oncogenic signalling within a narrow range (the ‘sweet spot’) that confers clones with a fitness advantage in specific permissive tissues [51, 52, 53]. This sweet spot is likely to vary across tissues, resulting in tissue-dependent mutational landscapes of the same protein (*e.g.*, KRAS) or proteins related to the same pathways (*e.g.*, other RAS isoforms, ERBB signalling, and RAS-dependent pathways).

In the first instance, a deeper understanding of cell signalling could lead to improved strategies for patient stratification (*e.g.*, a specific mutation in a specific tissue might require a specific therapeutic approach). Moreover, therapeutic interventions do not always eradicate all cancer cells, resulting in disease relapsing often with resistant or more aggressive tumours. Genetic heterogeneity of cancer, particularly in advanced disease, is undoubtedly a major contributor to therapeutic resistance. However, we are increasingly recognising how the plasticity of signalling pathways and cell-to-cell heterogeneity of non-genetic origin contribute significantly to limiting the efficacy of clinical interventions [54,55]. Cell signalling can indeed induce transitions to cellular states refractory to therapy, either before or in response to treatment [32,56, 57, 58]. Moreover, current pharmacological interventions aim to alter the activity of specific nodes in biochemical networks. However, feedback mechanisms (*e.g.*, reviewed in the study by Roesch et al. [32]) within the target pathway or other related signalling networks often compensate for the loss of signalling induced by inhibitors.

Arguably, the elucidation of mechanisms underpinning the development of an organism, tissue homeostasis, pathogenesis, and management of disease strongly depends on our capability to understand cell signalling and cell-to-cell variability.

## Perspectives and conclusions

Since the advent of modern cell biochemistry, the scientific community has made immense progress in understanding cell signalling and cellular decisions. However, traditional tools for cell biochemistry offered limited spatial and temporal resolution, for example, through cell fractionation and snapshot detection of biochemical activities at different times with immunoblotting.

We have illustrated how cell signalling is a dynamic process that evolves in space and time at different scales [59], from the fast dynamics of molecular interactions, occurring in seconds and tens of minutes required by immediate response genes, and minutes to days required by biological responses; from nanometer-scale conformational changes of signalling proteins, the propagation of signals between cellular compartments to macroscopic cell-to-cell communication mediated by paracrine and endocrine mechanisms.

For these reasons, fluorescence microscopy has been an invaluable tool to deepen our understanding of cell signalling as it provides low invasiveness, high spatiotemporal and biochemical resolutions, and high specificity, complementing other techniques. Nowadays, we have an ever-growing palette of biosensors (Figure 3b) at our disposal that we can use to probe the activity of biochemical pathways (*e.g.*, ERK, PI3K, AMPK), second messengers (*e.g.*, cAMP, calcium concentration), and metabolic networks (*e.g.*, ATP, lactate, pyruvate concentrations) in single living cells and tissues [60••, 61, 62]. The use of biosensors is somewhat established and gradually reaching an increasing number of non-specialist laboratories. Integrating biochemical imaging with microfluidics also facilitates the study of cell signalling with real-time control of both stimuli and responses [63, 64•, 65, 66, 67••, 68].

Notably, optogenetics (Figure 3c) – the capability to control biochemical reactions by light – is proving to be extremely powerful in probing biochemical networks [69, 70, 71, 72]. For example, Wilson et al. [34] have demonstrated the dynamic and combinatorial control of genes by ERK using OptoSOS to stimulate RAS signalling. OptoSOS is a fusion of light-inducible heterodimers (Figure 3c) that facilitate the translocation of SOS as a cargo protein to the plasma membrane. The guanine nucleotide exchange factor SOS then activates RAS proteins. At the same time, the authors monitored the activation of ERK by the nuclear translocation of a fluorescently tagged ERK; the transcription of target genes using an engineered sequence within the nascent RNA that induces the localisation in sites of transcription of a second fluorescent protein; and the expression of the target protein tagged with a third genetically encoded fluorophore. Other innovations in fluorescence microscopy, such as high-content imaging, multiplexed biochemical imaging are also laying the foundation for systems level understanding of cell decisions [34,55,73, 74, 75, 76, 77•, 78].

Moreover, research in the study of cell-to-cell communication and how cell signalling regulates the collective behaviour of cells is intensifying [79••, 80, 81]. For example, with the use of a light-inducible RAF kinase and a FRET-based sensor for ERK activity (see also Figure 3), Aoki et al. [81] characterised waves of ERK signalling that depend on ADAM17 travelling opposite to the direction of collective movement of cells. Using genetically engineered cells that express the inducible oncogene BRAF^V600E^ and translocation-based biosensors, Aikin et al. [82] have demonstrated a switch in autonomous signalling dynamics of mutant cells, that trigger a paracrine ADAM17-dependent wave of ERK signalling in its neighbourhood leading BRAF^WT^ cells to migrate towards the mutant cell. These efforts have to be matched by computational tools to aid the analysis of imaging data permitting us to monitor biochemical activities and cell fates in populations of living and interacting cells [83, 84, 85•, 86, 87, 88, 89, 90, 91, 92, 93, 94]. Further methodological innovations might be necessary, for example, in computational biology to integrate data at multiple scales and to model the emerging properties of cell populations.

Cell signalling can be described as a network of networks operating across cellular compartments and between cells. With constantly improving technologies, such as genomic editing, biochemical imaging, single-cell sequencing, *in vitro* organ-like cultures, and machine learning, we might soon be able to reveal how spatiotemporal dynamics of interconnected biochemical networks in a tissue cooperate to maintain both cellular and tissue homeostasis, how cell signalling might change during ageing, and how disease often stems from the corruption of cell signalling.

## Conflict of interest statement

Nothing declared
